# Psychosocial Burden and Quality of Life of Surveyed Nurses during the SARS-CoV-2 Pandemic

**DOI:** 10.3390/ijerph20020994

**Published:** 2023-01-05

**Authors:** Katarzyna Tomaszewska, Bożena Majchrowicz, Katarzyna Snarska, Beata Guzak

**Affiliations:** 1 Department of Nursing, Institute of Health Protection, The Bronisław Markiewicz State Higher School of Technology and Economics, 37-500 Jarosław, Poland; 2 Department of Nursing, Institute of Health Protection, State Academy of Applied Sciences, 37-700 Przemyśl, Poland; 3 Department of Clinical Medicine, Medical University of Bialystok, 15-089 Białystok, Poland; 4 Center for Postgraduate Education of Nurses and Midwives, Warsaw Medical University, 02-106 Warsaw, Poland

**Keywords:** stress, occupational burnout, quality of life, nurses, COVID-19

## Abstract

This study analyzes the impact of occupational burnout on the quality of life (QOL) of nurses surveyed during the SARS-CoV-2 pandemic. A total of 668 active nurses employed in public hospitals in Podkarpackie voivodeship (Poland) were surveyed. Throughout the pandemic, all wards where responders worked had a division into so-called “clean” and “dirty” zones, as well as balanced working hours. The research used the authors’ survey questionnaire Maslach Burnout Inventory (MBI) and the Polish version of the World Health Organization Quality of Life Instrument Short Form (WHOQOL-BREF). Descriptive statistics were used in the analysis of the collected material, while correlations between ordinal or quantitative variables were made using Spearman’s-rho coefficient. According to 94.0% of respondents, stress is an integral part of the nursing profession. The mean of the respondents’ MBI burnout was 50.83 +/− 9.05 pts. The respondents’ overall quality of life also averaged 65.74 +/− 13.12 pts. There were negative statistically significant correlations between the MBI and BREF domains, most of which were characterized by clear strengths of association. Higher exhaustion in various occupational aspects is associated with poorer quality of life in individual domains.

## 1. Introduction

Exposure to unknown diseases, especially pandemics, increases the risk of occupational burnout among nurses [1]. Previous studies by other authors have shown that COVID-19 had a significant negative impact on the mental health of healthcare workers, including stress, anxiety, depression, burnout, post-traumatic stress symptoms, and sleep disorders [2]. As the prevalence of COVID-19 infection increases, nurses’ occupational burnout worsens [3,4]. Occupational burnout is a state of physical and mental exhaustion resulting from chronic exposure to occupational stress or physically demanding work-related conditions [5]. The dangers of working with patients during the SARS-CoV-2 pandemic, the fear of infection, the unpredictability of events, the feeling of helplessness, and the anxiety of performing existing job duties are just some of the elements that nurses currently face while working. Therefore, the mental health of nurses working with patients infected with COVID-19 must be monitored and maintained during an outbreak. The services provided will only be of high quality if the work environment provides nurses with the right conditions to support them [6].

Nurses often face tremendous psychological pressure as a result of overwhelming workloads, long hours, shift duties, and working in high-risk environments [7]. This leads to occupational burnout, which is characterized by a sense of emotional exhaustion and a decline in feelings of competence and productivity at work [6,8,9,10,11,12]. As defined by Maslach and Jackson, occupational burnout is a psychological syndrome characterized by emotional exhaustion, depersonalization, and a reduced sense of professional efficacy [13,14]. The dimensions of this negative psychological reaction include overwhelming exhaustion, feelings of cynicism and detachment from work, and a sense of ineffectiveness and lack of achievement. The individual experience of stress in a social context is the most relevant factor [15]. The potential consequences of burnout for patients and nurses are serious and can include, among other things, decreased productivity, resulting in poor quality of care and increased medical errors and adverse events, poor patient safety, lower job satisfaction, and increased intent to leave the profession [9]. The causes of burnout can be traced to three areas: individual (age, gender, education, marital status, low psychological resilience, low self-esteem, lack of security or satisfaction with personal life), interpersonal (employee–client relationship, inability to achieve a balance between care for self and others, rivalry, psychological violence, bullying) and organizational (work overload, low wages, lack of PPE and poor working conditions). The above-mentioned terms may or may not be conducive to occupational burnout. It all depends mainly on the individual characteristics of the employee and the situation itself [16]. Social professions, in which interpersonal contact plays an important role, are primarily prone to the emergence of occupational burnout. Ongoing research on occupational burnout among nurses shows that it is a common phenomenon that occurs in this group of healthcare workers. Many researchers address this issue and describe its effects on nurses’ health, as well as on the quality of their duties, cooperation in the interdisciplinary team, and work organization [17]. Systematic reviews by a number of authors have found that nurses experience high levels of burnout during the COVID-19 pandemic, which is influenced by sociodemographic, social, and professional factors [18,19]. The psychological problems of nurses are not only stress or bad emotions. Depressive states, insomnia, and anxiety are very common among them. About 50.4% of nurses admitted to having depressive episodes. Feelings of anxiety caused by an increase in stressful situations were experienced by up to 90.0%, which had a significant impact on the development of professional burnout [20].

Quality of life (QOL) is an important concept in the fields of health and medicine. QOL is a complex concept that is interpreted and defined differently within and across disciplines, including health and medicine [21]. The prolonged COVID-19 pandemic has caused work overload for nurses and high-stress levels. Improving the quality of life, including professional life, can be a useful mediator for these demands [22]. Working in this profession is both stressful and demanding, which can put nurses at risk and affect their quality of life. Poor quality of life can affect the quality of services that nurses are required to provide to their patients [23]. Nurses are often exposed to difficult situations in the clinical area that can negatively affect their quality of life. Therefore, being optimistic and proactive can help nurses develop a positive attitude toward life, improve overall health, achieve high longevity and low-stress levels, and gain effective coping skills [24].

Observing the prevalent workload and elevated stress levels among nurses during the SARS-CoV-2 pandemic, we decided to study the impact on the phenomenon of occupational burnout and the quality of life (QOL) of the respondents.

## 2. Materials and Methods

### 2.1. Research Design

In the present study, a survey was conducted among nurses working with patients infected with the SARS-CoV-2 virus in public hospital wards in Podkarpackie voivodeship in Poland. All respondents performed their professional duties when surveyed. Throughout the pandemic, all wards where the respondents worked had a division into so-called “clean” and “dirty” zones, as well as balanced working hours. In addition, all zones for COVID-19 patients had intensive care stations. It was assumed that nurses with a seniority of more than five years would be surveyed, so that work experience would include work before the pandemic. This is the only way to determine whether factors affecting the occurrence of burnout syndrome have increased or not. Nurses employed in emergency departments were excluded from the study. The survey was conducted between February and May 2022. The epidemic state was canceled in Poland two weeks after the survey was completed.

### 2.2. Research Tools

The first part of the research tool was a survey questionnaire of the authors’ own authorship containing a total of 11 questions on sociodemographic data and the specifics of working with COVID-19 patients. The second part consisted of a standardized Maslach Burnout Inventory (MBI) questionnaire and a Polish version of the World Health Organization Quality of Life Instrument Short Form (WHOQOL-BREF). The Maslach Burnout Inventory (MBI) was developed in 1981 by Ch. Maslach and S.E. Jackson [25]. The test evaluates three aspects of burnout syndrome: emotional exhaustion, depersonalization, and a reduced sense of self-fulfillment. It consists of 22 test questions assessing the frequency of the aforementioned aspects on a 0–6 point scale, divided into three subscales relating to each aspect of burnout itself. Responses are given according to a 7-point frequency scale, where 0 means “never” and 6 means “every day.” The score is calculated separately for each subscale, adding up the points for each aspect: emotional exhaustion—high (>27), moderate (17–26), low (0–16); depersonalization—high (>13), moderate (7–12), low (0–6); lack of achievement—high (0–31), moderate (32–38), low (>39). The higher the score on the emotional exhaustion and depersonalization scales, the more intense the burnout is, and the lower the score on the sense of self-fulfillment, the higher the burnout rate.

Quality of life was assessed using the Polish version of the World Health Organization Quality of Life Instrument Short Form (WHOQOL-BREF) in four domains: physical, psychological, social, and environmental. The WHOQOL-BREF consists of 26 questions. Each aspect of quality of life was rated on a 5-point scale (very bad, bad, neutral, good, very good). The questionnaire contained several questions that were analyzed separately: question 1 is about the overall individual perception of quality of life, and question 2 is about the overall individual perception of health. Domain scores reflect individual perceptions of the QOL domains and have a positive direction—the higher the score, the higher the QOL. The overall score for each domain is calculated by counting the average of all the items included in each domain [13]. Participation in the study was anonymous and voluntary.

### 2.3. Participants

The study group consisted of 668 nurses employed in hospital wards in Podkarpackie voivodeship in Poland, recruited by non-probabilistic sampling. Each respondent independently and voluntarily completed the survey questionnaire and gave written consent to participate in the study, and each respondent received information about the processing of the respondents’ personal data. The consents and survey questionnaires are in the possession of the author of the paper. All distributed questionnaires were accepted and completed. Initially, 750 questionnaires were distributed, and 668 were accepted and correctly completed, accounting for 89.6%. The inclusion criterion for the study was working with patients infected with the SARS-CoV-2 virus and work experience as a nurse for a minimum of 5 years. The exclusion criterion was a lack of consent to participate in the study, work experience for less than five years, and no professional contact with COVID-19 patients. The questionnaires were left in one of the nursing rooms and, after completion, were personally collected by the authors of the study.

### 2.4. Statistical Analysis

In the analysis of the collected material, descriptive statistics were used to describe the most important information about the variables analyzed in the study and the study group. Correlations between ordinal or quantitative variables (during the unfulfilled conditions of using parametric tests) were made using Spearman’s-rho coefficient, which indicates the intensity of the relationship and its direction, i.e., positive or negative. The resulting values ranged from −1 to 1, with −1 indicating a perfect negative correlation and 1 a perfect positive correlation. The analysis was performed using the IBM SPSS 26.0 package with the Exact Tests module. All correlations and differences are statistically significant when *p* ≤ 0.05.

### 2.5. Ethical Procedures

The participation of nurses in the study was voluntary and anonymous. The study was conducted in accordance with the ethical standards set forth in the Declaration of Helsinki (64th WmA General Assembly, Fortaleza, Brazil, October 2013) and in accordance with Polish legal regulations. The study was approved by the Bioethics Committee of the State Eastern European University in Przemyśl (KBPWSW No. 03/2022).

## 3. Results

The study was designed to analyze the phenomenon of occupational burnout, stress levels, and quality of life (QOL) for nurses during the COVID-19 pandemic. A total of 688 nurses were surveyed in the Podkarpackie voivodeship. The characteristics of the study group are shown in Table 1.

According to 90.8% of the surveyed nurses working with COVID-19-positive patients, workstations were fully equipped with personal protective equipment. A total of 89.0% of respondents claimed that staffing shortages were caused by positive COVID-19 test results with a referral to isolation or quarantine. The majority of respondents stated that all procedures were followed when working with COVID-19 patients, such as balanced working hours (57.3%) or access to personal hygiene products and bathrooms with showers (80.1%).

For 27.0% of respondents, working during the pandemic caused an increase in the level of perceived stress. Contact with patients infected with the SARS-CoV-2 virus caused fear and anxiety about their immediate families. A total of 90.0% of respondents reported that the isolation and inability of patients to contact their families caused them to expect more attention and time from nursing staff, which resulted in the experience of emotional tension and stressful situations due to frequent staff shortages. The stress level of 45.5% of respondents was at a moderate level. Only 7.0% of the respondents were thinking about changing their profession.

In our own research, the mean of the respondents’ MBI burnout was 50.83 +/− 9.05 pts. The minimum value of burnout was 25 pts., while the maximum value was 78 pts. The mean of emotional exhaustion of the nurses surveyed was at a high level and was 23.9 +/− 5.66 pts. The average score of depersonalization was 10.75 +/− 2.73 pts. and the average score of a reduced sense of self-fulfillment was 16.17 +/− 4.19 pts. Detailed results are presented in Table 2.

BREF scores were standardized on a scale from 0 to 100, with 100 representing the best quality of life and zero representing the worst. The overall quality of life for the respondents was at an average level of 65.74 +/− 13.12 pts. The average quality of life in the physical domain of the respondents was 62.13 +/− 17.86 pts., in the psychological domain 67.24 +/− 20.83 pts., in the social domain 69.77 +/− 17.09 pts., and in the environmental domain 63.84 +/− 18.75 pts. Detailed results are presented in Table 3.

There are negative statistically significant correlations between the MBI and BREF domains, most of which are characterized by clear strengths of association (higher exhaustion in various professional aspects is associated with poorer quality of life in each domain). Considering the overall MBI score and the overall BREF score, it can be claimed that higher professional exhaustion is associated with poorer quality of life. The correlation, in this case, is statistically significant and shows a significant strength of association (Table 4).

More than a dozen statistically significant correlations were shown between age, education, job tenure, occupational burnout, and quality of life, but all of them were characterized by very weak strengths of association. Taking into account the level of perceived stress and MBI and BREF, more pronounced correlations were observed (all correlation coefficients between stress scores and MBI and BREF proved statistically significant). The results show that higher levels of perceived stress are associated with higher overall professional burnout, higher emotional exhaustion, and a poorer quality of life. The results are presented in Table 5.

## 4. Discussion

The presented study analyzed the impact of the COVID-19 pandemic on the incidence of stress and occupational burnout phenomena, as well as on the quality of life (QOL) of surveyed nurses. 

It was demonstrated that up to the day of the survey, the level of burnout of MBI respondents averaged 50.83 +/− 9.05 pts. The scores of individual subscales were also at a moderate level. The overall quality of life of the nurses surveyed on a scale from 0 to 100 was at a moderate level and was 65.74 +/− 13.12 pts. For 27.0% of the respondents, work during the pandemic had become more stressful. The level of stress in 45.5% of respondents was at a moderate level. Only 7.0% of the respondents thought about changing their profession. According to other authors, there is a link between stress and burnout or chronic fatigue and burnout. Burnout can develop as a chronic reaction to stress [26,27,28,29]. Occupational burnout is also associated with declining mental health among nurses and poorer quality of patient care and is thus a significant problem in healthcare delivery [30]. An analysis by Slusarz et al. found that the highest burnout scores were reported among nurses working during the COVID-19 pandemic [31], which is confirmed by other studies [32,33]. In addition to factors such as a poor working environment, heavy workload, and low wages, occupational burnout can affect nurses’ quality of life (QOL).

In our own study, the overall quality of life of respondents was at an average level of 65.74 +/− 13.12. The average quality of life in the physical domain of respondents was 62.13 +/− 17.86, in the psychological domain 67.24 +/−20.83, in the social domain 69.77 +/− 17.09, and in the environmental domain 63.84 +/− 18.75, which confirmed the studies of other authors [34,35]. Different results were obtained in a Canadian study, where the vast majority of respondents reported symptoms of stress, and 22% intended to leave their current employment [36]. Additionally, in other studies, job satisfaction, job stress, and turnover intention were factors affecting the quality of work life. Work stress and psychosocial risks are associated with increased employee absenteeism, a loss of productivity, and high healthcare costs. As a result, positive factors, such as job satisfaction, had stronger effects than negative factors [37], as confirmed by the studies of other authors [38,39,40,41].

Publications by other authors demonstrate that nurses caring for patients with COVID-19 reported lower quality of life in the social domain; in addition, nurses who had susceptible individuals in their families reported poorer physical quality of life [42,43]. In our own study, despite perceived stress, the quality of life in each domain was at a moderate level. Other studies have shown that social support and a sense of coherence are significant predictors of high quality of life in all domains [44,45,46,47].

The results of other authors’ studies showed that 60% of nurses said they had a moderate level of quality of work life. A significant relationship was found between sociodemographic variables and the quality of work-life score. No significant differences were observed between the nurses’ quality of work-life scores with employment status, salary, age, gender, and marital status [48,49].

In our own study, higher levels of perceived stress were associated with higher overall professional burnout, higher emotional exhaustion, and poorer quality of life, which confirmed the results of other authors’ studies [50]. The results of other own studies have shown that nurses working with COVID-19 patients are exposed to various stressors leading to professional burnout and that the conditions of working with a COVID-19-positive patient are related to perceived stress [6,51].

### Limitations of the Study

The survey was conducted among a group of nurses employed in healthcare facilities over a certain period of time, which means that the results of the survey and its conclusions cannot be generalized. Nurses provided subjective opinions, and current psychological well-being influenced the respondents’ assessments of the situation. In addition, there was an opportunity to exchange opinions among respondents during the survey. At the same time, among the surveyed group before the pandemic, there were no studies on the level of perceived stress, as well as the phenomena of occupational burnout and quality of life. It is necessary to conduct further multi-center studies to generalize the results and implement recommendations for management.

## 5. Conclusions

The survey of hospital-employed nursing staff providing services in units for patients infected with COVID-19 provides insights into the impact of the SARS-CoV-2 pandemic on nurses’ stress levels and occupational burnout.

The results of our own study prove that working conditions with COVID-19-positive patients have a moderate association with experiencing symptoms of occupational burnout and quality of life in all its domains. Additional measures should be developed and implemented to reduce the incidence of burnout among this professional group. Moreover, Poland is a country with a shortage of nursing staff. The task of the management should be to implement procedures and educational measures to prevent the occurrence of this phenomenon among nurses and, at the same time, to create attractive working conditions that allow young citizens to become interested in this profession. 

## Figures and Tables

**Table 1:** Characteristics of the study group.

Variable		Frequency (*n* = 688)	
Gender	Female	654	95.1%
	Male	19	2.8%
Age (years)	20–30	90	13.1%
	31–40	112	16.3%
	41–50	204	29.7%
	>51	282	41.0%
Education *	Medical high school	118	17.2%
	Post-secondary medical school	111	16.1%
	Bachelor of Science in Nursing	238	34.6%
	Master of Science in Nursing	221	32.1%
Marital status	Married	526	76.5%
	Single	101	14.7%
	Divorced	32	4.7%
	Widowed	29	4.1%
Work experience (years)	5	86	12.5%
	6–10	68	9.9%
	11–20	124	18.0%
	>21	410	59.6%

* Until 2000, in Poland, pre-graduate education for nurses took place in a 5-year medical high school after graduating from an 8th grade elementary school or a 2/2.5-year post-secondary medical school after graduating from medical high school. Since 2000, pre-graduate education for nurses and midwives has been conducted in a two-degree university system: nurses are awarded a Bachelor’s degree in nursing after 3 years, and a Master’s degree in nursing after the next 2 years.

**Table 2:** Mean value of MBI occupational burnout of surveyed nurses.

	Frequency (*n*)		Average	Median	Standard Deviation	Minimum	Maximum
	Valid	Invalid					
MBI Total (22–88 pts.)	688	0	50.83	51.00	9.05	25.00	78.00
Emotional exhaustion (9–36 pts.)	688	0	23.91	24.00	5.66	9.00	36.00
Depersonalization (5–20 pts.)	688	0	10.75	11.00	2.73	5.00	20.00
Reduced sense of self-fulfillment (8–32 pts.)	688	0	16.17	16.00	4.19	8.00	32.00

**Table 3:** Mean scores of WHOQOL-BREF quality of life scale of surveyed nurses.

	Frequency (*n*)		Average	Median	Standard Deviation	Minimum	Maximum
	Valid	Invalid					
Physical area (0–100 pts.)	688	0	62.13	60.71	14.94	17.86	100.00
Psychological area (0–100 pts.)	688	0	67.24	66.67	14.53	20.83	100.00
Social area (0–100 pts.)	688	0	69.77	75.00	17.09	0.00	100.00
Environmental area (0–100 pts.)	688	0	63.84	65.63	13.92	18.75	100.00
BREF Total (0–100 pts.)	688	0	65.74	67.08	13.12	17.04	100.00

**Table 4:** Correlation results between MBI occupational burnout and WHOQOL-BREF quality of life domains.

Spearman’s rho		MBI Total (22–88 pts.)	Emotional Exhaustion (9–36 pts.)	Depersonalization (5–20 pts.)	Reduced Sense of Self-Fulfillment (8–32 pts.)
Physical area (0–100 pts.)	Correlation coefficient	−0.529	−0.529	−0.234	−0.314
	Significance (two-tailed)	0.000	0.000	0.000	0.000
	Frequency (*n*)	688	688	688	688
Psychological area (0–100 pts.)	Correlation coefficient	−0.556	−0.485	−0.324	−0.365
	Significance (two-tailed)	0.000	0.000	0.000	0.000
	Frequency (*n*)	688	688	688	688
Social area (0–100 pts.)	Correlation coefficient	−0.409	−0.357	−0.225	−0.283
	Significance (two-tailed)	0.000	0.000	0.000	0.000
	Frequency (*n*)	688	688	688	688
Environmental area (0–100 pts.)	Correlation coefficient	−0.418	−0.374	−0.194	−0.314
	Significance (two-tailed)	0.000	0.000	0.000	0.000
	Frequency (*n*)	688	688	688	688
BREF Total (0–100 pts.)	Correlation coefficient	−0.554	−0.500	−0.282	−0.377
	Significance (two-tailed)	0.000	0.000	0.000	0.000
	Frequency (*n*)	688	688	688	688

**Table 5:** Influence of sociodemographic data and perceived stress levels on MBI and WHOQOL-BREF quality of life burnout levels.

Spearman’s rho		Age	Education	Job Seniority	On a Scale of 1 to 5, How Would you Rate the Level of Stress Experienced When Working with SARS-CoV-2 Infected Individuals?
MBI Total (22–88 pts.)	Correlation coefficient	0.045	−0.078 *	0.062	0.324 **
	Significance (two-tailed)	0.243	0.042	0.106	0.000
	Frequency (*n*)	688	688	688	682
Emotional exhaustion (9–36 pts.)	Correlation coefficient	0.128 **	−0.073	0.188 **	0.312 **
	Significance (two-tailed)	0.001	0.054	0.000	0.000
	Frequency (*n*)	688	688	688	682
Depersonalization (5–20 pts.)	Correlation coefficient	0.068	−0.107 **	0.023	0.193 **
	Significance (two-tailed)	0.074	0.005	0.548	0.000
	Frequency (*n*)	688	688	688	682
Reduced sense of self-fulfillment (8–32 pts.)	Correlation coefficient	−0.133 **	−0.012	−0.146 **	0.166 **
	Significance (two-tailed)	0.000	0.749	0.000	0.000
	Frequency (*n*)	688	688	688	682
Physical area (0–100 pts.)	Correlation coefficient	−0.118 **	0.124 **	−0.130 **	−0.280 **
	Significance (two-tailed)	0.002	0.001	0.001	0.000
	Frequency (*n*)	688	688	688	682
Psychological area (0–100 pts.)	Correlation coefficient	−0.052	0.113 **	−0.075	−0.296 **
	Significance (two-tailed)	0.174	0.003	0.051	0.000
	Frequency (*n*)	688	688	688	682
Social area (0–100 pts.)	Correlation coefficient	−0.085 *	0.134 **	−0.111 **	−0.243 **
	Significance (two-tailed)	0.026	0.000	0.004	0.000
	Frequency (*n*)	688	688	688	682
Environmental area (0–100 pts.)	Correlation coefficient	−0.007	0.125 **	−0.026	−0.228 **
	Significance (two-tailed)	0.862	0.001	0.503	0.000
	Frequency (*n*)	688	688	688	682
BREF Total (0–100 pts.)	Correlation coefficient	−0.073	0.144 **	−0.095 *	−0.301 **
	Significance (two-tailed)	0.055	0.000	0.013	0.000
	Frequency (*n*)	688	688	688	682

** Correlation significant at the 0.01 level (two-tailed). * Correlation significant at the 0.05 level (two-tailed).

## Data Availability

The datasets used and analyzed during the current study available from the corresponding author on reasonable request.
